# Comprehensive Physicochemical Analysis of Polyphosphate-Modified Alginate Matrices: Synthesis, Structural Analysis, and Calcium Ion Release Dynamics

**DOI:** 10.3390/ma18051114

**Published:** 2025-02-28

**Authors:** Alicja Wawszczak, Magdalena Czemierska, Anna Jarosz-Wilkołazka, Dorota Kołodyńska

**Affiliations:** 1 Department of Inorganic Chemistry, Institute of Chemical Sciences, Faculty of Chemistry, Maria Curie-Skłodowska University, M. Curie Skłodowska Sq. 2, 20-031 Lublin, Poland; 2 Institute of Biological Sciences, Maria Curie-Skłodowska University, Akademicka 19, 20-031 Lublin, Poland; magdalena.czemierska@mail.umcs.pl (M.C.); anna.jarosz-wilkolazka@mail.umcs.pl (A.J.-W.)

**Keywords:** alginates, internal cross-linking, polyphosphate, porosity

## Abstract

The selection of cross-linking techniques is essential for the development of the alginate matrix. In this study, we investigated porous sodium alginate matrices (ALG1@in, ALG3@in, ALG5@in) synthesized by internal gelation and further functionalized with polyphosphate (PP) at concentrations of 5% and 15% (ALG3@inPP5, ALG3@inPP15). Extensive characterizations were conducted, employing scanning electron microscopy coupled with energy-dispersive spectroscopy (SEM-EDS) for morphological and compositional analysis, Fourier transform infrared spectroscopy (FTIR-ATR) for structural elucidation, thermogravimetric analysis (TGA-DTG) for thermal stability, and porosimetry (ASAP) for surface area and pore size evaluation. Surface charge density (pH_ZPC_) was determined, and Ca^2^⁺ release kinetics were monitored in demineralized water over 7 days and Dulbecco’s phosphate-buffered saline (DPBS) over 14 days. The increase in sodium alginate concentration increases the BET surface area and pore volume, which improves adsorption and transport properties. The thermal stability of the tested matrices at 37 °C confirms their suitability for biomedical applications. The ALG3@in sample showed the best parameters, combining high BET surface area (11.02 m^2^/g), significant pore volume (0.08 cm^3^/g) and thermal stability up to 257 °C, making it a suitable candidate for applications in biology, tissue engineering and processes requiring sterilization and high temperatures. These findings underscore the potential of polyphosphate modifications to improve alginate matrices, opening avenues for future applications in areas like cell culture scaffolds or environmental chemistry solutions.

## 1. Introduction

In recent years, biopolymer porous sodium alginate matrices have become a popular issue addressed by scientists in various fields. Among them, matrices based on calcium alginate have attracted great interest. Calcium alginate is widely regarded as a versatile natural polysaccharide due to its excellent biocompatibility and non-toxic nature. These properties have made it one of the preferred materials for the development of platforms, carriers, and matrices used in environmental, agricultural, and biomedical applications. Obtaining these types of materials requires a precise and careful selection of synthesis parameters and appropriate cross-linking methods [1,2,3].

Alginate consists of blocks of (1 → 4)-linked β-D-mannuronate (M) and α-L-guluronate (G) residues, the ratio and arrangement of which significantly influence the gelling properties [4]. Depending on the method used, the designed materials will differ in terms of mechanical strength, porosity, and stability.

The most frequently chosen method of cross-linking alginate is ionic cross-linking using calcium chloride as an external cross-linking method or calcium carbonate as an internal cross-linking method [5].

The external cross-linking process using calcium chloride involves contacting a sodium alginate solution which results in the binding of Ca^2+^ with the carboxyl site instead of Na^+^ in the sodium alginate, resulting in immediate gelation [6]. Each Ca^2+^ ion attaches to the COO^−^ in sodium alginate because of strong electrostatic attraction [7].

The internal cross-linking, also known as in situ gelation, involves dissociating a sparingly soluble calcium salt such as a calcium carbonate in alginate solution and adding an acidifying agent, which gradually lowers the pH, thus leading to the release of Ca^2+^ ions, which interact with the alginate chains, forming cross-links. The biggest advantage of internal cross-linking is its gradual process, which allows for greater control, which is particularly beneficial in the case of encapsulation. However, this method requires precise control of the concentration and degree of dispersion of calcium salts and the acidifying agent. Moreover, it is a much slower process and leads to difficulties in obtaining a high degree of cross-linking [8,9].

The porous sodium alginate matrices are increasingly subjected to various modifications in order to improve their physicochemical properties. Numerous reports have been published in the literature regarding the modification of alginate with phosphates [10,11,12,13], highlighting its potential to improve mechanical properties, stability, and bioactivity [14]. However, while there are some studies that discuss the modification of the alginate structure using polyphosphate, the results have often been inconsistent, and critical analyses, such as comprehensive physicochemical characterizations and in-depth biological assessments, are still lacking [15]. This gap underscores the need for more rigorous and systematic investigations to fully understand the effects of polyphosphate modification on alginate matrices.

This study involved the synthesis of porous matrices using sodium alginate (ALG1@in, ALG3@in, ALG5@in), followed by a comprehensive analysis employing techniques such as scanning electron microscopy (SEM), Fourier transform infrared spectroscopy with attenuated total reflectance (FTIR-ATR) and thermogravimetric analysis with derivative thermogravimetry (TGA-DTG).

Furthermore, alginate matrices were modified with polyphosphate (PP) at varying concentrations, specifically 5% (ALG3@inPP5) and 15% (ALG3@inPP15) relative to the mass of sodium alginate used. The modified matrices have been comprehensively analyzed by scanning electron microscopy with energy dispersive spectroscopy (SEM-EDS), Fourier transform infrared spectroscopy (FTIR-ATR), acceleration surface area and porosimetry (ASAP) and water content. The surface charge density (pH_ZPC_) was also measured. Furthermore, the Ca^2+^ ion release profile was monitored over a period of 7 days in demineralized water (DW) and over 14 days in Dulbecco’s phosphate-buffered saline (DPBS) solution. We intend to describe the techniques and to comment on the results obtained after polyphosphate modification for obtaining materials useful for developing polymer matrices prepared for cell cultures.

## 2. Materials and Methods

### 2.1. Chemicals

The materials and chemicals used in this research are as follows: sodium alginate derived from brown algae (Carl Roth, Karlsruhe, Germany, Cat. No. 9180.2) used as the primary gel-forming polymer; calcium carbonate (POCH, Cat. No. 878330111) served as a source of calcium ions, functioning as a cross-linking agent for the alginate gels; glucono-δ-lactone (≥99.0%, Sigma-Aldrich, St. Louis, MO, USA, Cat. No. G4750-500G) acted as an acidifying agent, facilitating the hydrolysis of calcium carbonate and ensuring a controlled release of calcium ions; sodium polyphosphate (Graham’s salt) (Merck, Rahway, NJ, USA, Cat. No. 1.06529.1000) was incorporated into the matrices as an additive. The cross-linking process was carried out following the method described by Gila-Vilchez et al. [16] ensuring effective gel formation and swelling behaviour. Additionally, sodium hydroxide and nitric acid (Chempur, Piekary Śląskie, Poland, Cat. No. 118109252 and 145296032, respectively) were used to adjust the pH of solutions to optimize the swelling behaviour. All chemicals were used as received without further purification.

### 2.2. Synthesis and Modification of Alginate Matrices

Porous alginate matrices were prepared using the internal (abbreviated as ‘in’) cross-linking method. Solutions of sodium alginate (ALG) with concentrations of 1, 3, 5 wt% (ALG1, ALG3, ALG5) were prepared by mixing ethanol with sodium alginate and then adding demineralized water. The solutions were mixed using a magnetic stirrer until the alginate was completely dissolved at a temperature not exceeding 40 °C. Calcium carbonate CaCO_3_ was dispersed in the calcium alginate solution and glucono-δ-lactone (GDL) was added and then poured into the prepared round molds. The synthesis was carried out maintaining the ratio of ALG:CaCO_3_:GDL equal to 1:0.15:0.533 g. The molds with solutions were left for 24 h until complete cross-linking to obtain (ALG1@in, ALG3@in and ALG5@in), then rinsed with demineralized water, frozen and freeze-dried. The synthesis of modified matrices with polyphosphate (PP) at 5% and 15% by mass of ALG proceeded similarly to that of conventional matrices; however, only sodium alginate at a concentration of 3% (*w*/*v*) was used to obtain ALG3@inPP5 and ALG3@inPP15. The subsequent steps of the procedure were analogous. The internally cross-linked matrices were subjected to freezing using a ULTF-37i low-temperature freezer (BitBlu Frost, Suszec, Poland) and then to freeze drying using an Alpha 1–2 LSC lyophilizator (Martin Christ GmbH, Osterode am Harz, Germany). During the activities carried out, a freezer was used under conditions of −80 °C for 24 h. Lyophilization was performed at −50 °C; 0.045 mBar (from 5 mBar) for 2 days. Before polyphosphate modification, it was necessary to determine the length of the polyphosphate chain, which was carried out according to the procedure described in Section 2.3.

### 2.3. Physicochemical Characterization

#### 2.3.1. Determination of Polyphosphate Chain Length

The polyphosphate structure determination was performed in accordance with the methodology described by Robinson et al. in [17]. The chain length of polyphosphates was determined using acid-base titration. The samples were prepared by dissolving 0.5 g of polyphosphate in 450 mL of deionized water. Titration was carried out using a 907 Titrando titrator paired with an 800 Dosino dosing unit (Metrohm, Herisau, Switzerland), a combined pH electrode, and a magnetic stirrer (model 801). The pH was adjusted to 2.5 using 1 M HCl, and the total volume was brought to 500 mL. The solution was divided into two portions: one was immediately titrated, while the other was hydrolyzed under reflux for 6 h to convert the polyphosphate to orthophosphate before titration. Titration was carried out by adding 0.1 M NaOH in 100 μL increments while recording the pH after each addition.

#### 2.3.2. Morphological Analysis

Morphology was examined using scanning electron microscopy (SEM, Quanta 3D FEG, FEI, Hillsboro, OR, USA). The surfaces of the samples were covered with a thin layer of platinum–gold to improve electrical conductivity. Images were recorded at different magnifications with a focus on the analysis of the surface and longitudinal sections. Subsequently, images were captured under an acceleration voltage of 5 kV, maintaining a specified working distance that falls within the range of 8.5 to 10.3 mm.

#### 2.3.3. FTIR-ATR Spectrometry

The Fourier transformation infrared (FTIR) spectra were acquired using Cary 630 FTIR with the ATR mode (Agilent, Santa Clara, CA, USA) conducted within the wavelength range of 500–4000 cm^−1^. Recording of the spectra was performed by the Micro Lab FTIR software, version B.04. Post-collection spectral analysis was carried out using the Agilent Resolutions Pro software, version 5.2.0.861.

#### 2.3.4. Thermogravimetric Analysis (TGA)

Thermogravimetric analysis (TGA) was performed on a TA Instruments Q50 (TA Instruments, Eden Prairie, MN, USA) in a nitrogen atmosphere. Samples were heated from 25 °C to 1000 °C at a rate of 10 °C/min. On the basis of the TG and DTG curves, characteristic temperatures for degradation processes were determined, including the maximum degradation rate temperature (T_max_).

#### 2.3.5. Textured and Structural Properties

The textural properties of the samples were determined by the nitrogen adsorption–desorption method using the ASAP 2420 apparatus (Micromeritics, Norcross, GA, USA). Samples weighing approximately 0.1735 g were degassed at 353.15 K, and measurements were taken at 77.35 K. Based on the adsorption–desorption isotherms, the specific surface area was determined using the BET method, the pore volume, and their size distribution.

#### 2.3.6. Water Content Determination

Using a moisture analyzer, the water content of the resulting dry matrices was determined using a moisture analyzer OHAUS MB25 (OHAUS Europe GmbH, Greifensee, Switzerland) at 378 K. By this procedure, a sample of the analyzer matrix was placed onto the pan and heated under controlled conditions. The weight of the sample was continually measured by the moisture analyzer as it was heated, enabling real-time estimation of the mass loss from evaporation of the water. The procedure was repeated until the sample reached a consistent weight, which denoted the removal of all water.

#### 2.3.7. Surface Charge Density (pH_ZPC_)

The point of zero surface charge (pH_zpc_) was determined on the pH changes in NaCl solutions (0.1 M) in contact with the matrices, which provided information on the ion exchange and hydrophilic properties of the surfaces. The pH at which a material’s net surface charge is zero is measured using this technique, and it is essential for understanding the surface chemistry of materials.

#### 2.3.8. Calcium Ions’ Release Profile

The method involved observing Ca^2+^ ions release from samples in two different solvents over set periods. Samples were prepared and submerged in demineralized water (DW) and Dulbecco’s phosphate-buffered saline (DPBS). The process was monitored for 7 days in demineralized water and 14 days in DPBS to assess both short-term and long-term release dynamics. These media were chosen for their varying complexity: DW for its purity, and DPBS for its physiological relevance. Analysis of the amount of calcium ions in solutions was carried out using the inductively coupled plasma optical emission spectrometry technique using the 720-ES ICP-OES (Varian, Palo Alto, CA, USA). Calcium was determined at a wavelength of 393.366 nm.

#### 2.3.9. Chemical Composition Analysis

The chemical composition of the samples was studied by X-ray photoelectron spectroscopy (XPS) (Prevac, Rogów, Poland) using monochromatic Al Kα radiation (1486.6 eV). Calcium ions content was analyzed by inductively coupled plasma emission spectrometry (ICP-OES, 720-ES ICP-OES, Varian). Calcium was determined at a wavelength of 393.366 nm. For this purpose, the samples were dissolved in a 2% sodium citrate solution overnight, then diluted and analyzed. X-ray diffraction XRD was used to determine the mineral composition. The analysis was performed using the powder method and the Panalytical X’pert PROMPD X-ray diffractometer with the PW 3050/60 goniometer in the angle range 2θ of 5–65 (Malvern Panalytical, Malvern, UK). As the X-ray source, a copper lamp Cu (CuK = 0.154178 nm) was applied. The X’ Pert High Score software (version 3.0e (3.0.5)) processed the diffraction data.

## 3. Results

### 3.1. SEM Analysis of ALG1@in, ALG3@in, and ALG5@in

The morphological features of ALG1@in, ALG3@in, and ALG5@in samples were analyzed by the SEM method to understand their structural differences. Detailed evaluation of the morphological analysis is shown in Table 1.

It was observed that all samples exhibit a porous structural morphology characterized by typical sponge. In the case of ALG1@in, the most compact morphology is observed. It is characterized by simple, smooth structures that indicate a less complex pore network. ALG3@in exhibits a more intricate pore structure, featuring irregular shapes and an increased number of interpore connections, which contributes to a higher structural complexity. ALG5@in with the highest concentration of 5% alginate presents a dense pore network. This network consists of smaller and more uniformly distributed pores, suggesting a significant influence of alginate concentration on the internal structure and density of the cross-linked matrices.

These findings highlight the impact of alginate concentration on the internal morphology and structural properties of cross-linked sodium alginate matrices.

The modification of alginates with polyphosphates (PPs) has gained significant attention due to its potential in enhancing osteogenic activity and promote bone regeneration. PP provides essential phosphoric acid units, which can facilitate the mineralization of calcium phosphate, a key process in bone formation, tissue engineering, and regenerative medicine [14,18].

### 3.2. Determination of the Length of the Polyphosphate Chain

In order to evaluate the quality of the polyphosphates utilized and guarantee their suitable characteristics throughout the modification procedure, it was essential to determine their average chain length.

**Figure 1:**
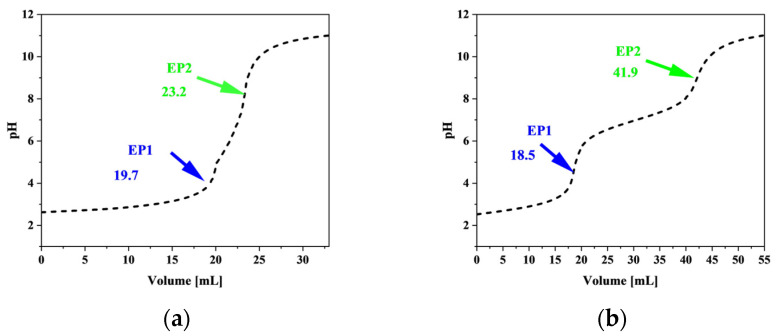
Titration curves of polyphosphate (**a**) before and (**b**) after hydrolysis.

Two titration curves were generated: one for the native polyphosphate and another for its hydrolyzed orthophosphate counterpart. Polyphosphates exhibited two equivalence points because of the presence of strongly and weakly dissociating protons, while cyclic phosphates showed only one equivalence point, reflecting their lack of end groups. The titration curves that illustrate these equivalence points are presented in Figure 1. The values (*f_o_*) and (*f_p_*) represent the difference in NaOH volume between equivalence points, where (*f_o_*) is measured for hydrolyzed orthophosphate and (*f_p_*) for polyphosphate. The average chain length (*n*) was calculated using the ratio of NaOH volumes required to neutralize orthophosphate (*f_o_*) and polyphosphate (*f_p_*) according to Equation (1).

(1) $$n=\frac{2\cdot f_{o}}{f_{p}}$$

The calculated average chain length (*n*) of the polyphosphate sample was found to be 13.08, based on titration data. The result indicates a predominance of medium-length linear polyphosphate species in the sample.

### 3.3. SEM Analysis of ALG3@in, ALG3@inPP5, and ALG3@inPP15

When the PP solution was added to the calcium alginate complexes, there was an interaction of PO_4_^3−^ ions with the ALG1@in, ALG3@in and ALG5@in complexes to nucleate the calcium salt due to the effects of supersaturation [6]. The EDS analysis of ALG3@in, ALG3@inPP5, and ALG3@inPP15 samples provides valuable information on the impact of the addition of PP on the chemical composition and potential changes in the structure of the alginate matrices (Table 2). The value of the carbon content systematically decreases with the increase in the addition of PP (the addition of PP potentially reduces the share of the organic part of alginate in favour of increasing the content of other elements). The oxygen content remains relatively constant after addition of PP, which may be related to the strong oxygen nature of both alginate and polyphosphate. A slight increase in the sodium content is observed as the concentration of PP in the matrix increases.

The calcium content increases from 2.49% for ALG3@in, through 2.06% for ALG3@inPP5, to 3.45% for ALG3@inPP15. This increase, especially noticeable with a higher PP content, may suggest intensification of the cross-linking process or the formation of more complex ionic structures with calcium ions. The appearance of phosphorus with the addition of polyphosphate is expected and increases from 0.76% for ALG3@inPP5 to 2.37% for ALG3@inPP15. The presence of phosphorus indicates the incorporation of polyphosphate into the structure. While SEM observations suggest a potential dispersion of phosphates within the alginate matrix, the current data are insufficient to conclusively confirm homogeneous distribution. PP is characterized by the linear structure composed of several orthophosphate monomer units connected by high-energy phosphoric anhydride bonds. Divalent cations such as Ca^2+^ are ionically cross-linked with linear PP [19].

The results of the EDS elemental composition analysis showed differences in the Ca/P molar ratio between the tested samples. For the ALG3@inPP5 sample, the ratio is 2.08, while for the ALG3@inPP15 sample it is 1.13. These values indicate different calcium and phosphorus contents in the samples. This may be due to different interactions of the matrix elements with calcium and phosphate ions during the forming process.

### 3.4. FTIR-ATR Analysis

**Figure 2:**
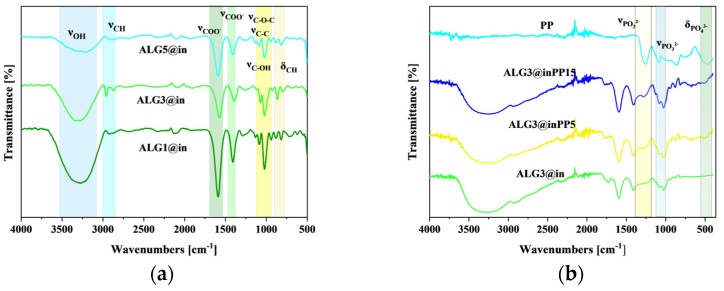
FTIR-ATR spectra of (**a**) ALG1@in, ALG3Qin and ALG5@in; (**b**) modified by PP alginate ALG3@inPP5 and ALG3@inPP15.

Information about the occurrence of specific functional groups was provided by spectroscopic analyses. The recorded spectra for all samples are presented and compared in Figure 2. Typical regions for the FTIR-ATR analysis of alginates are presented in Table 3.

The analysis of the spectra showed the presence of important functional groups for alginate, including hydroxyl and carboxyl groups. In the range of 3600–2800 cm^−1^, stretching vibrations of the OH bonds occur. In the case of internally cross-linked matrices, the stretching vibrations of aliphatic C-H are much more distinctive, observed at 2970–2850 cm^−1^. The bands present at 1585 and 1410 cm^−1^ were assigned to the asymmetric and symmetric C-O stretching vibrations in the COO^−^ groups, respectively. At 1086 and 1018 cm^−1^, the vibrations were identified as stretching for C-O-C (cyclic ether) and C-C, respectively.

In the case of internally cross-linked matrices, the band at 1130 cm^−1^ occurs. A characteristic of uronic acid stretching vibration was detected at 935 cm^−1^ with the participation of C-CH and C-O-H deformations. Due to the use of smaller amounts of calcium ions compared to sodium alginate in ALG1@in, ALG3@in and ALG5@in, more intense peaks are observed for internally cross-linked matrices. This means that in the polysaccharide structure there are more free carboxyl and hydroxyl groups, and the composition of calcium alginates has a significant effect on the growth rate of calcium phosphates. Similar results were observed in [6].

After modification by PP, a band shift from 1585 to 1594 cm^−1^ and from 1410 to 1413 cm^−1^ is observed in the higher wavenumber. The observed band shift suggests the formation of a chemical bond between the mineral phase and the organic matrix; i.e., the interaction between the positive charge of calcium and the negative charge of the carboxyl/phosphate groups. A new strong absorption band can also be observed at 552 cm^−1^, which is attributed to the bending of PO_4_^3−^. As presented in [21], the intense peaks located at 1030, 605, and 563 cm^−1^ are assigned to PO_4_^3−^. However, it is well known that sodium alginate also has a strong absorption band at this place. Thus, the observed stretching band at 1035 cm^−1^ is attributed to the overlap of C-O-C and PO_4_^3−^. Therefore, the presence of intermolecular interactions between inorganic minerals and alginate chains in the polymer network enhances the structural organization and stability of the material, influencing its mineralization properties [22].

### 3.5. TGA-DTA Analysis

Thermogravimetric analysis (TGA) was performed to quantitatively evaluate the extent of incorporation of calcium carbonate within the synthesized specimens. The application of calcium carbonate and glucono-δ-lactone (GDL) is a one-step cross-linking method used to obtain ALG samples, which leads to a slow gelation process of polymers [23]. The prepared formulations were evaluated for the impact of the cross-linking agent on the pharmaceutical properties of ALG. This analysis also answered the question as to whether the observed carbonates originated from atmospheric absorption or if they constituted residues of calcium carbonate unaltered by hydrolytic processes. The thermogravimetric analysis was conducted to assess the weight loss behaviour of samples under a heating rate of 25 °C to 1000 °C at a rate of 10 °C/min.

**Figure 3:**
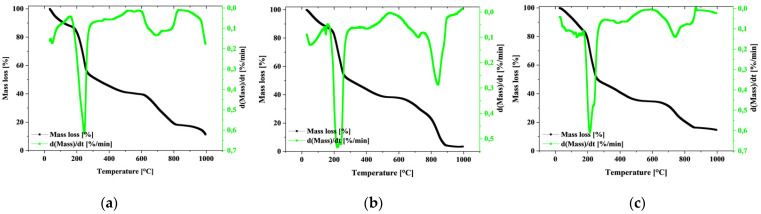
TGA and DTG curves of alginate-based samples with different ALG concentrations synthesized by internal cross-linking: (**a**) ALG1@in, (**b**) ALG3@in, (**c**) ALG5@in.

As presented in Figure 3, the alginate-based samples with different ALG concentrations synthesized by internal cross-linking typically exhibit reduced thermal stability (broader and stronger DTG peaks), which could be a sign of increased bound water release during heating or weaker cross-linking interactions with calcium carbonate.

Higher sodium alginate concentration matrices appear to be more thermally stable, which could indicate better cross-linking and a denser polymer network.

The first low temperature peak (generally below 200 °C) corresponds to the loss of water physically bound in the matrix. This is a dehydration phenomenon that is relatively independent of alginate concentration [24]. This stage differs significantly depending on the type of cross-linking. High peaks in the temperature range around 200 °C to 400 °C may be related to the decomposition of more complex aqueous complexes and chemically bonded dehydration, and they may also be the beginning of the breakdown of the polysaccharide network [25]. Subsequent mass decreases in the TGA graphs and peaks in the DTG at higher temperatures, often between 400 °C and 600 °C, indicate more intense degradation of the material, including the breakdown of polysaccharide chains and the loss of other organic components. Peaks in the range of the highest temperatures, close to 800 °C, may indicate the degradation of the remaining alginate components or pyrolysis reactions, where the chemical structure undergoes a profound change with the formation of carbon (carbonization process) [26]. In detail, for the ALG1@in simple, the first mass loss (5%) is observed in the temperature range of 0–64 °C. The subsequent degradation stages include a mass loss of 39% in the range of 210–260 °C and 22% in the higher temperature range, from 635 to 788 °C. The peak values on the DTG curve for ALG1@in are 246 °C, 558 °C and 693 °C, indicating the occurrence of three main stages of thermal degradation. For the ALG3@in matrix, 5% mass loss occurs in the range of 0–71°C. The main degradation, which accounts for 40% of the mass loss, occurs in the range of 196–257 °C. The next phase, including 25% mass loss, occurs in the range of 806–873 °C. The DTG curve shows several degradation peaks: the first two at 134 °C and 148 °C, the next at 220 °C and 242 °C, and three more at 394 °C, 719 °C and 843 °C. For the ALG5@in matrix, the first mass loss (5%) occurs in the range of 0–83 °C. The next stages include a mass loss of 39% at temperatures of 195–246 °C and 19% in the range of 702–810 °C. The peaks in the DTG curve occur at 213 °C, 385 °C and 741 °C, suggesting a more gradual degradation process compared to other concentrations.

### 3.6. Water Content

The water content in the ALG3@in sample is much higher (23%) than in the other samples. The ALG3@inPP5 sample shows a water content of 7.3%. This is a much lower value compared to ALG3@in, suggesting that the addition of 5% PP reduces the retention of water in the matrix. The ALG3@inPP15 sample has the lowest water content (6.8%). PP can increase the degree of cross-linking of the alginate matrix through additional ionic bonds with Ca^2+^ ions. A high concentration of PP, as in the ALG3@inPP15 sample, leads to the formation of a more compact and densely cross-linked structure, which limits the availability of water. This results in less water retention, despite the hydrophilicity of the polyphosphate itself. In the case of the ALG3@inPP15 sample, the structures are stabilized by interactions between the PP and the polymer matrix. These interactions can reduce the free space in the matrix that could be occupied by water, thus reducing its content. The addition of PP can reduce the elasticity and increase the stiffness of the matrix, which limits its ability to absorb water. The increased compactness and stiffness of the matrix make it more difficult to retain water in the structure, which is especially visible in the ALG3@inPP15 sample.

### 3.7. pH_ZPC_ Analysis

**Figure 4:**
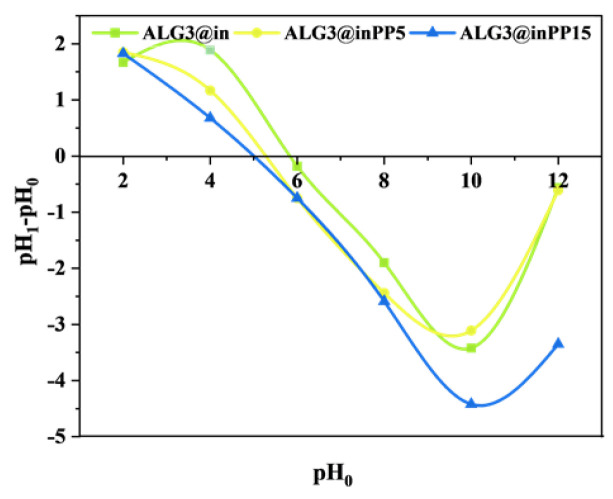
The relationship between initial pH (pH_0_) and the change in pH (pH_1_–pH_0_) for ALG3@in, ALG3@inPP5, and ALG3@inPP15.

The determination of the surface charge density as a function of pH in a 0.1 M NaCl solution for externally cross-linked matrices revealed that the surface charge becomes increasingly negative with increasing pH levels (Figure 4). At pH values from 5.9, a negative charge emerges on the surface of the ALG3@in, ALG3@inPP5 and ALG3@inPP15 matrices and the magnitude of this charge depends on the amount of PP added. Specifically, a greater addition of PP results in a lower pH threshold at which the negative charge manifests [27]. It is well known that the way that matrices interact with cells, proteins, and medications is impacted by surface charge alteration. A positive charge enhances protein adsorption, which stimulates proliferation, and encourages cell adhesion through electrostatic interactions with negatively charged cell membranes [28]. A negative charge enhances binding to positively charged compounds, limiting nonspecific protein adsorption [29,30].

### 3.8. XRD Analysis

**Figure 5:**
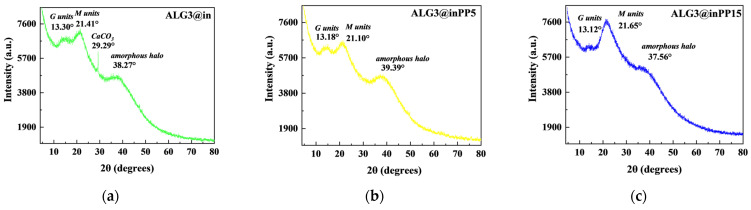
XRD patterns of (**a**) ALG3@in, (**b**) ALG3@inPP5, and (**c**) ALG3@inPP15 samples.

For ALG3@in, the diffraction peaks at 2θ values of approximately 38.27°, 21.41° and 13.30° are connected with the sodium alginate (corresponding to the (110) plane of the polyguluronate unit, the (200) plane of the polymannuronate unit) (Figure 5). After modification by PP for ALG3@inPP5 and ALG3@inPP15 samples, the diffraction peaks correspond to the 2θ values at 39.39°, 21.10° and 13.18° as well as 37.56°, 21.65° and 13.12°. A peak connected with calcium carbonate (ref. code 01-080-2801) was also found. It was speculated that the structure was caused by the combination of calcium ions on ALG3@in and phosphate ions.

### 3.9. XPS Analysis

**Figure 6:**
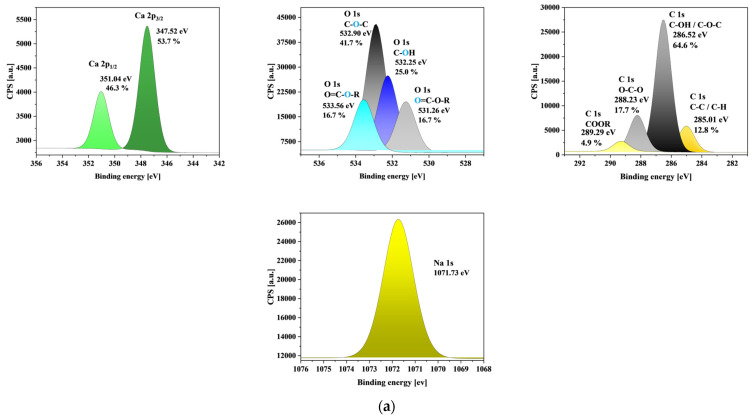

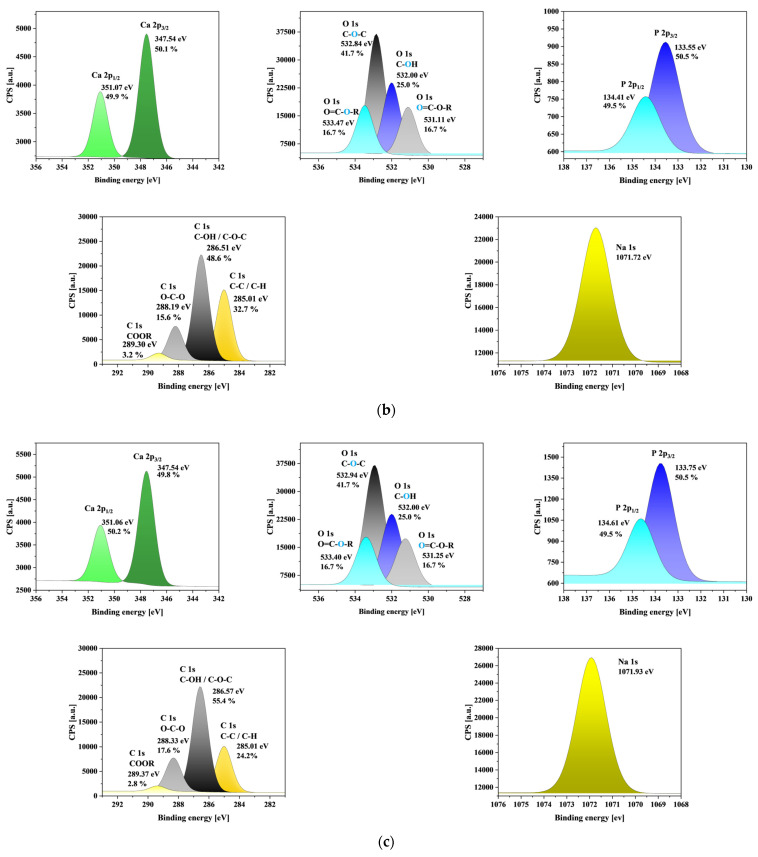
XPS high-resolution spectra of (**a**) ALG3@in, (**b**) ALG3@inPP5, and (**c**) ALG3@inPP15 samples.

**Figure 7:**
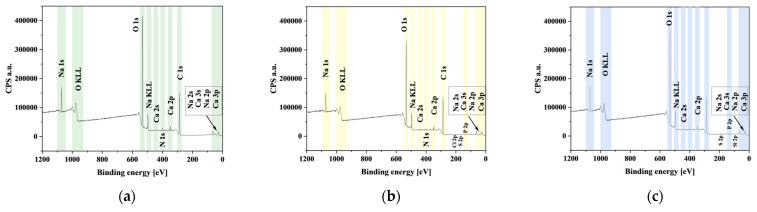
XPS survey scan spectra of (**a**) ALG3@in, (**b**) ALG3@inPP5, and (**c**) ALG3@inPP15 samples.

The XPS spectra for ALG3@in, ALG3@inPP5, and ALG3@inPP15 are compared in Figure 6. Additionally, the XPS survey peaks for the samples studied are described in Table 4. The XPS fast scan survey spectrum (Figure 7) of ALG3@in, ALG3@inPP5, and ALG3@inPP15 suggests the existence of sharp peaks of C 1s, O 1s, Na 1s, and Ca 2p at 286.52 eV, 532.90 eV, 1071.73 eV, and 347.52 eV for ALG3@in; sharp peaks of C 1s, O 1s, Na 1s, Ca 2p, and P 2p at 286.51 eV, 532.84 eV, 1072.72 eV, 347.54 eV, and 133.55 eV for ALG3@inPP5; and C 1s, O 1s, Na 1s, Ca 2p, and P 2p at 286.57 eV, 532.94 eV, 1071.93 eV, 347.54 eV, and 133.75 eV, respectively. The chemical atomic contents of O, P, and Ca are 43.6%, 0%, and 1.1% for ALG3@in; 36.6%, 0.7%, and 1.0% for ALG3@inPP5; and 41.1%, 1.8%, and 1.2% for ALG3@inPP15, respectively. The data suggest that P is incorporated into the surface composition of ALG3@inPP5 and ALG3@inPP15 after modification.

Four peaks were detected for the C 1s signal and come from the C-C, C-O-C, C-OH, and -COO groups. The surface oxygen content indicates the formation of four different O-bonds. The first two peaks were identified for the O 1s signal and can be assigned to the C-O-C, C-OH and C-O groups. As for the P 2p spectrum, two peaks were detected. They indicate the existence of two types of P-bonds at 133.55 eV and 134.41 eV, which suggests the formation of PO_4_^3−^ and P-O groups. Two peaks found from the deconvoluted bands of the O 1s region at 533.47 and 531.11 eV for ALG3@inPP5 as well as at 533.47 and 531.11 eV for ALG3@inPP15 could be assigned to P=O and P-OH, respectively [31,32].

### 3.10. Calcium Ions’ Release Profile

**Figure 8:**
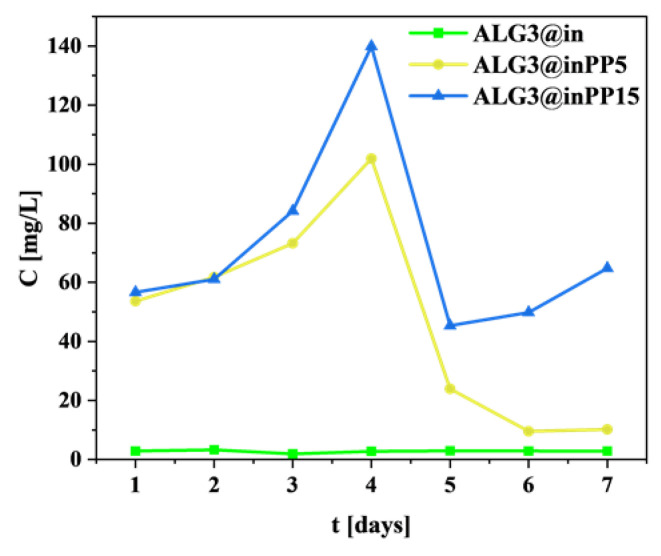
Ca^2+^ ions’ release profile for ALG3@in, ALG3@inPP5, and ALG3@inPP15 in DW after 7 days.

The ALG3@in sample shows the lowest concentration of released Ca^2+^ ions, practically unchanged throughout the entire test period (Figure 8).

Numerous biological advantages, such as improved osteoblast proliferation, differentiation, and mineralization of the extracellular matrix, are provided by controlled Ca^2+^ release in bone and tissue regeneration [33]. Furthermore, calcium ions are critical for efficient tissue regeneration because they stimulate angiogenesis and modulate the inflammatory response [34]. The efficacy of biomaterials in clinical applications is enhanced by the ability to control Ca^2+^ release, allowing for greater control over the regeneration process [35].

**Figure 9:**
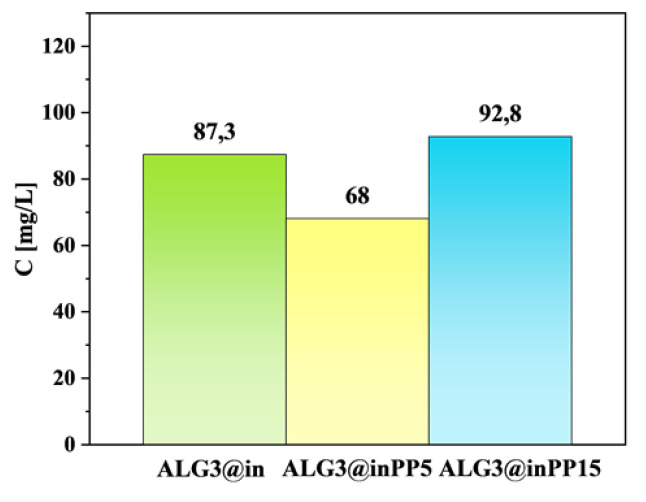
Total amount of Ca^2+^ ions’ release profile for ALG3@in, ALG3@inPP5, and ALG3@inPP15 in DPBS buffer after 14 days.

The maximum concentration is approximately 10 mg/L, suggesting that calcium carbonate cross-linking in the presence of GDL is highly effective. A low level of Ca^2+^ ions release may indicate good structural stability of the sample in DW. ALG3@inPP5 shows a significantly higher level of Ca^2+^ ions’ release. Their concentration increases to approximately 90 mg/L on day 4, followed by a sharp decline to approx. 20 mg/L on day 7. The initial increase is due to the release of Ca^2+^ ions from sites unbound or weakly bound in the structure. ALG3@inPP15 shows an even higher release profile, reaching a maximum concentration of approximately 140 mg/L also on day 4. After this time, there is a sharp decrease in Ca^2+^ ions’ concentration, although on day 7 a certain increase in concentration is visible. PP by forming complexes with Ca^2+^ ions may influence their release. In addition, they are unstable in aqueous solution and undergo hydrolysis, which may lead to the breakdown of these complexes and the release of Ca^2+^ ions into the solution. In the DPBS buffer and ALG3@inPP15 sample, a higher concentration of PP may accelerate the hydrolysis process, leading to faster breakdown of calcium–polyphosphate complexes. Hydrolysis of these complexes releases Ca^2+^ ions into the solution, resulting in a larger amount of Ca^2+^ in the solution compared to the ALG3@inPP5. The higher concentration of PP in the ALG3@inPP15 sample may also affect the cross-linking structure of the alginate matrix. A larger number of polyphosphates can lead to more branched and looser network structures, increasing the permeability of the matrix and allowing for easier release of Ca^2+^ ions. The total amount of Ca^2+^ released in DPBS buffer after 14 days is presented in Figure 9.

### 3.11. Accelerated Surface Area and Porosimetry

Table 5 presents the results of the analysis of the BET specific surface area, pore volume, micropore volume and average pore diameter of ALG1@in, ALG3@in, and ALG5@in, as well as ALG3@inPP5 and ALG3@inPP15.

**Figure 10:**
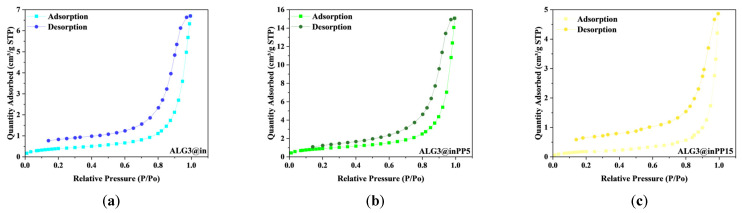
The N_2_ adsorption/desorption isotherm plot of matrices of (**a**) ALG3@in, (**b**) ALG3@inPP5, and (**c**) ALG3@inPP15.

The ALG3@inPP5 sample shows the highest specific surface area (3.32 m^2^/g), and ALG3@inPP15 the lowest (0.66 m^2^/g). ALG3@inPP5 has the largest pore volume (0.02 cm^3^/g), while ALG3@inPP15 has the smallest (0.01 cm^3^/g). No significant micropore volume was observed in all samples. The average pore diameter ranges from 13.95 nm (ALG3@inPP15) to 15.24 nm (ALG3@inPP5). Figure 10 demonstrates the N_2_ sorption isotherms of ALG3@in, ALG3@inPP5, and ALG3@inPP15. The samples do not exhibit any obvious sorption hysteresis loop, indicating its dense structure and low specific surface area.

The addition of polyphosphates (PPs) clearly reduces the specific surface area and pore volume, especially in the case of ALG3@inPP15. A high PP15 content can lead to a more compact and dense matrix structure, resulting in less surface area available for gas adsorption and smaller pore volume. The ALG3@inPP5 sample, despite the addition of PP, maintains a relatively high specific surface area and pore volume, which suggests that a moderate amount of polyphosphates (5%) allows for optimal cross-linking without a significant reduction in porosity. ALG3@inPP15, with a higher PP concentration, shows significantly reduced specific surface area and pore volume, which is a consequence of the more compact matrix structure, which may affect its adsorption and mechanical properties. The adsorption and desorption isotherms shown in the graphs for the ALG3@in, ALG3@inPP5, and ALG3@inPP15 samples exhibit characteristics typical of mesoporous materials. This hysteresis, which resembles the shape of the H3 type, suggests the presence of irregular or fissured pores, in which the process of capillary condensation during adsorption and pore emptying during desorption occur with a delay. The highest adsorption capacity was observed for the ALG3@inPP5 sample, where the amount of adsorbed gas reaches a maximum value of about 16 cm^3^/g, indicating the presence of the most developed pore structure. Compared to it, the ALG3@in sample shows a much lower adsorption capacity, reaching about 6–7 cm^3^/g, indicating a smaller number of available pores or their limited volume. In contrast, the ALG3@inPP15 sample shows the lowest adsorption capacity, not exceeding 5 cm^3^/g, suggesting reduced pore availability or pore blockage due to specific structural changes.

## 4. Discussion

Calcium alginate matrices are highly valued for their biocompatibility, non-toxicity, and suitability for various fields such as environmental, agricultural, and biomedical applications. One of the key advantages of calcium alginate matrices is their ability to be tailored for specific needs through the careful selection of synthesis parameters and cross-linking methods. Ionic cross-linking, primarily using calcium chloride or calcium carbonate, is a common approach to form these matrices.

Modifying the physicochemical properties of alginate matrices is a growing area of interest, and the modification of ALG3@in with polyphosphate (PP), e.g., ALG3@inPP5 and ALG3@inPP15, has demonstrated potential benefits. It can be applied particularly in bone regeneration or to develop polymer matrices for cell culture.

In this study, alginate matrices were synthesized using different concentrations of sodium alginate, followed by internal cross-linking with calcium carbonate CaCO_3_. The matrices were further modified by adding PP at varying concentrations (5% and 15%). Detailed characterization using SEM-EDS, FTIR-ATR, TGA, and ASAP revealed significant differences in the structure and chemical composition of the matrices after PP modification. The Ca/P ratio values obtained in the tested samples suggest differences in the possible behaviour of these materials because the solubility of calcium phosphate materials strongly depends on the Ca/P ratio and the crystalline phases present. The lower the Ca/P ratio, the higher the potential solubility [36]. The presence of calcium-rich phases, which may exhibit lower solubility and longer stability, for example, in the physiological environment, is connected with the ALG3@inPP5 sample, which has a Ca/P ratio equal to 2.08. On the other hand, the ALG3@inPP15 sample (Ca/P = 1.13) has a composition that favours greater bioresorbability. This may support biomineralization processes and the controlled release of calcium and phosphate ions.

SEM analysis showed that alginate matrices with higher alginate concentrations exhibited denser pore networks, while the addition of PP altered the chemical composition by increasing calcium and phosphorus content, which indicated enhanced cross-linking.

FTIR-ATR spectra provided insight into the interactions between the alginate and PP, suggesting that the addition of PP led to shifts in key absorption bands, indicating new chemical bonds formed between calcium ions and the carboxyl/phosphate groups. These shifts, particularly in the C–O and P–O regions, suggest that polyphosphate not only modifies the physical properties of the alginate matrix but also promotes mineralization, enhancing the material’s bioactivity. This chemical interaction is further supported by XPS analysis, which confirmed the presence of phosphorus in the modified matrices, validating the successful incorporation of PP.

Thermogravimetric analysis (TGA) provided information on the thermal stability of the modified matrices. It was observed that the alginate matrices with higher concentrations of PP such as ALG3@inPP15 exhibited lower thermal stability compared to unmodified alginates, suggesting that PP modification may affect the matrix’s resistance to heat degradation. Additionally, the water retention capacity of the matrices was significantly reduced with higher polyphosphate content, likely due to the increased cross-linking and reduced porosity, which limited the availability of water within the matrix.

ALG3@in and ALG5@in exhibit higher BET surface area and larger pore volume compared to ALG1@in, which may be crucial in applications requiring efficient adsorption, such as drug carriers, biosensors, and bioactive substance delivery systems in biomedical engineering. The thermal stability of samples ALG1@in (up to 260 °C), ALG3@in (up to 257 °C), and ALG5@in (up to 246 °C) indicates their suitability in sterilization processes and in technologies requiring stability at elevated temperatures, such as catalyst carriers or filter materials. Under biological conditions (37 °C), all samples remain stable; however, the larger surface area and pore volume of ALG3@in increase its potential as a cell culture material, where gas exchange and nutrient transport are essential. ALG3@in, with the highest BET surface area and developed mesoporous structure, is the most optimal for further modification, offering a balance between adsorption, mass transport, and thermal stability, making it a versatile candidate in biomaterial engineering and nanotechnology.

The Ca^2+^ release profiles demonstrated the influence of PP on the release kinetics. The modified matrices, especially those with higher PP concentrations, released higher amounts of Ca^2+^ ions. This phenomenon is particularly relevant in biomedical applications, where the controlled release of ions can be crucial for tissue regeneration and healing. The results also indicated that the release rate was influenced by the concentration of PP, with higher concentrations leading to faster release rates due to the increased number of complexes formed.

Surface charge density measurements revealed that the surface charge of the matrices became more negative as the pH increased, with higher PP concentrations leading to a more pronounced negative charge. This behaviour is indicative of the increased interaction between the phosphate groups and the alginate matrix, further enhancing the material’s bioactive potential. Moreover, BET surface area analysis showed that PP modification significantly reduced the specific surface area and pore volume, particularly in the case of ALG3@inPP15, which had the most compact and dense structure. This reduction in porosity may affect the adsorption capacity and mechanical properties of the matrices, particularly for applications requiring a high surface area for interactions with cells or drugs.

In summary, this study demonstrates that the modification of alginate matrices with PP not only improves the mechanical properties but also enhances the bioactivity of the materials, making them more suitable for applications in tissue engineering and bone regeneration. The findings provide valuable insights into the design of more effective biopolymer matrices for advanced biomedical applications.

## Figures and Tables

**Table 1:** SEM micrographs of alginate samples with different ALG concentrations, synthesized as internally cross-linked ALG1@in, ALG3@in and ALG5@in.

Mag.	100×	1000×	25,000×
ALG1@in	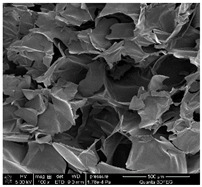	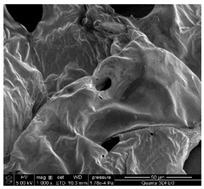	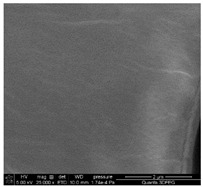
ALG3@in	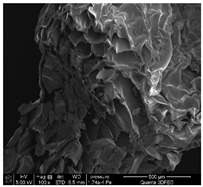	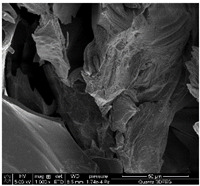	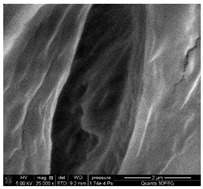
ALG5@in	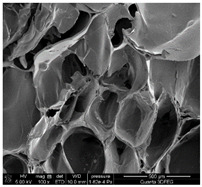	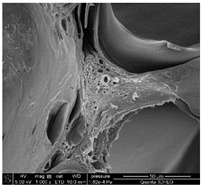	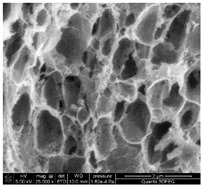

**Table 2:** SEM micrographs of alginate-based samples after polyphosphate modification of ALG3@in, ALG3@inPP5, and ALG3@inPP15.

	Element	wt%	at%	SEM Image
ALG3@in				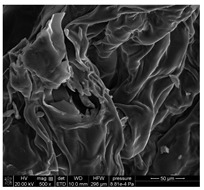
	C K	36.76	45.03	
	O K	54.65	50.16	
	Na K	6.10	3.91	
	Ca K	2.49	0.91	
ALG3@inPP5				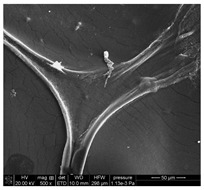
	C K	33.27	41.45	
	O K	55.83	52.16	
	Na K	8.07	5.25	
	P K	0.76	0.37	
	Ca K	2.06	0.77	
ALG3@inPP15				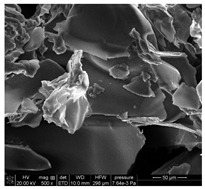
	C K	31.62	40.11	
	O K	55.46	52.63	
	Na K	7.21	4.78	
	P K	2.37	1.17	
	Ca K	3.45	1.32	

**Table 3:** Typical regions for the FTIR-ATR analysis of alginates [20].

Region	Characterization
3600–2800 cm^−1^	The stretching vibrations of CH and OH contribute to 100% of the potential energy distribution; for example, there is 3365 cm^−1^ in each spectrum, which is due to the OH stretching vibration of surface -OH groups in the materials, and CH stretch appears between 2990 and 2879 cm^−1^
1500–1200 cm^−1^	The local symmetry region mainly constitutes deformational vibrations of groups having a local symmetry, such as HCH, and the vibrations of the CH_2_OH group
1200–950 cm^−1^	A C-O stretching region, associated with C-O and C-C contributions, but the contribution from C-O to the potential energy distribution appreciably exceeds that from C-C
950–700 cm^−1^	The side groups’ deformational region (C-OH, C-CH, O-CH), which includes the important ‘fingerprint’ or anomeric bands between 930 and 840 cm^−1^, and an appreciable contribution from the stretching of C-C
Below 700 cm^−1^	The skeletal region, which could be split in two, namely, 700–500 cm^−1^ (a ‘crystalline region’ for the exocyclic CCO deformations is observed) and below 500 cm^−1^ (for the endocyclic CCO, CCC deformations)
Phosphate groups	The bands observed at 1033–1044 cm^−1^ are assigned to the asymmetric stretching mode of vibrations of the phosphate bonds P-O

**Table 4:** XPS analysis of ALG3@in, ALG3@inPP5, and ALG3@inPP15 samples.

Sample	Peak Component	Binding Energy [eV]	FWHM [eV]	Area/(RSF × T × MFP)	%At Conc	% St.Dev.
ALG3@in	C 1s	286.2	2.3	417,309.5	51.6	0.067
	N 1s	400.2	1.9	8792.9	1.1	0.073
	O 1s	533.0	2.5	352,885.6	43.6	0.061
	Na 1s	1071.5	2.0	21,540.2	2.7	0.018
	Ca 2p	347.7	1.8	8881.1	1.1	0.029
ALG3@inPP5	C 1s	286.8	3.1	432,873.1	58.1	0.071
	N 1s	400.8	2.2	6588.3	0.9	0.070
	O 1s	532.8	2.5	272,559.2	36.6	0.056
	Na 1s	1072.1	2.0	17,015.6	2.3	0.019
	P 2p	134.6	2.2	5461.5	0.7	0.041
	Ca 2p	347.6	1.7	7342.0	1.0	0.034
	S 2p	169.8	2.4	1754.9	0.2	0.022
	Cl 2p	200.6	3.3	1872.3	0.3	0.012
ALG3@inPP15	C 1s	286.7	2.6	392,955.6	50.3	0.066
	N 1s	399.9	2.2	13,285.6	1.7	0.047
	O 1s	532.7	2.6	321,024.7	41.1	0.059
	Na 1s	1071.9	1.9	22,396.1	2.9	0.018
	P 2p	134.4	2.2	13,698.4	1.8	0.038
	Ca 2p	347.4	1.9	9492.8	1.2	0.034
	S 2p	169.7	2.4	3915.4	0.5	0.021
	Si 2p	102.2	2.8	4059.3	0.5	0.035

**Table 5:** ASAP parameters of ALG1@in, ALG3@in ALG5@in, ALG3@inPP5, and ALG1@inPP15.

	ALG1@in	ALG3@in	ALG5@in	ALG3@inPP5	ALG3@inPP15
BET surface area [m^2^/g]	3.61	11.02	11.13	3.32	0.66
V [cm^3^/g]	0.02	0.08	0.07	0.02	0.01
V_micro_ [cm^3^/g]	0.000023	0.000165	0.000188	0.0000	0.0000
D [nm]	13.37	14.76	16.92	15.24	13.95

## Data Availability

The original contributions presented in this study are included in the article. Further inquiries can be directed to the corresponding authors.
